# The Effects of Different Types of Exercise on Pulmonary Inflammation and Fibrosis in Mice with Type 2 Diabetes Mellitus

**DOI:** 10.3390/cells14131026

**Published:** 2025-07-04

**Authors:** Haoyang Gao, Xiaotong Ma, Ze Wang, Danlin Zhu, Yifan Guo, Linlin Zhao, Weihua Xiao

**Affiliations:** 1 Shanghai Key Lab of Human Performance, Shanghai University of Sport, Shanghai 200438, China; haoyanggao1999@163.com (H.G.); maxiaotong_1026@163.com (X.M.); wz_arrebol@163.com (Z.W.); zdl981030@163.com (D.Z.); 2 School of Elderly Care Services and Management, Nanjing University of Chinese Medicine, Nanjing 210023, China; yifan_guo@njucm.edu.cn; 3 School of Physical Education, Shanghai Normal University, Shanghai 200234, China

**Keywords:** type 2 diabetes mellitus, exercise, diabetic lung disease, pulmonary fibrosis, pulmonary inflammation

## Abstract

**Background:** Diabetic lung disease, characterized by inflammation and fibrosis, is an emerging chronic complication of type 2 diabetes mellitus (T2DM). However, systematic studies on the effects of exercise interventions remain limited. This study aimed to investigate the impact of different exercise types (swimming, resistance training, and high-intensity interval training [HIIT]) on pulmonary inflammation and fibrosis in T2DM mice, and to explore underlying molecular mechanisms. **Methods:** A T2DM mouse model was established by a high-fat diet (HFD) combined with streptozotocin (STZ) induction. Mice were randomly divided into sedentary control, swimming, resistance training, and HIIT groups, and underwent 8 weeks of exercise intervention. After the intervention, body composition was assessed. Lung histopathological changes were evaluated by hematoxylin&eosin (HE) and Masson staining. Inflammatory cytokines, fibrosis markers, and the expression of the TGF-β1/Smad signaling pathway were detected. Macrophage infiltration and polarization were also analyzed. **Results:** Exercise intervention improved body composition and reduced oxidative stress in T2DM mice. All three exercise modalities downregulated inflammatory cytokine expression, inhibited macrophage activation and M1 polarization, and promoted M2 polarization. Additionally, exercise improved lung tissue structure, reduced collagen deposition, and decreased the expression of fibrosis-related markers. Furthermore, anti-fibrotic effects were mediated by suppression of the TGF-β1/Smad signaling pathway and inhibition of epithelial-mesenchymal transition (EMT). Among the interventions, HIIT demonstrated the strongest inhibitory effect on the TGF-β1/Smad pathway, while swimming showed the most significant anti-inflammatory benefits. **Conclusions:** Different types of exercise effectively alleviate pulmonary inflammation and fibrosis in T2DM mice. These effects are closely related to the inhibition of oxidative stress, regulation of macrophage polarization, and suppression of TGF-β1/Smad signaling activation, with swimming and HIIT demonstrating superior protective benefits.

## 1. Introduction

Diabetes mellitus (DM) is a chronic metabolic disease characterized by persistent hyperglycemia and has become a major global public health concern. A recent study reported that by 2022, approximately 828 million adults worldwide were living with DM, representing an increase of 630 million cases since 1990 [1]. Type 2 diabetes mellitus (T2DM), also referred to as non-insulin-dependent diabetes, constitutes the vast majority of cases, accounting for approximately 90–95% [2]. Persistent high blood glucose levels in T2DM can cause progressive damage to various organs, particularly the vascular and nervous systems, giving rise to complications such as diabetic nephropathy, cardiomyopathy, and retinopathy [3,4,5,6], which are major contributors to diabetes-related disability and death.

Approximately 50 years ago, Schuyler [7] first reported the impact of diabetes on pulmonary function. In recent years, accumulating evidence has demonstrated a significant association between diabetes and pulmonary dysfunction [8,9,10,11]. One study found that for each 1% increase in glycated hemoglobin levels, there is an estimated decline of 1.99% in forced vital capacity (FVC) and 1.04% in forced expiratory volume in one second (FEV1) [12]. Hyperglycemia, insulin resistance, chronic low-grade inflammation, and obesity are considered key contributors to pulmonary impairment in T2DM [13]. In addition to functional decline, structural alterations such as thickening of the alveolar epithelium and capillary basement membrane, chronic inflammation, and interstitial fibrosis have been identified in the lungs of diabetic patients [8,14,15,16]. The pathogenesis of pulmonary inflammation and fibrosis in T2DM involves multiple mechanisms, including oxidative stress [17], chronic inflammation [16], epithelial–mesenchymal transition (EMT) [18], and activation of the transforming growth factor-β (TGF-β)/Smad signaling pathway [19]. Furthermore, alveolar macrophages, as resident immune cells in the lungs, play essential roles in maintaining tissue homeostasis through involvement in extracellular matrix remodeling, angiogenesis, and immune responses. In T2DM, macrophage infiltration and activation in lung tissue lead to excessive release of pro-inflammatory and pro-fibrotic cytokines, exacerbating pulmonary inflammation and fibrosis [20]. Diabetes also increases the risk and worsens the prognosis of various respiratory diseases, including chronic obstructive pulmonary disease (COPD), pneumonia, and tuberculosis [21,22,23], making pulmonary complications a major factor affecting the quality of life and survival of diabetic patients.

Exercise is widely recognized as a safe, cost-effective, and multi-system intervention strategy, playing a key role in improving metabolic control, glucose homeostasis, and preventing or treating diabetes complications. Recent preclinical studies have shown that swimming, treadmill running, voluntary exercise, and high-intensity interval training (HIIT) can improve respiratory mechanics, suppress oxidative stress, chronic inflammation, apoptosis, and pathological changes in diabetic rodents [24,25,26,27]. Clinically, Kim et al. [12] reported that lung function decline is associated with poor glycemic control, and walking more than 300 min per week may help prevent pulmonary deterioration in diabetic patients. However, the effects of resistance training on pulmonary complications in T2DM have not yet been reported. While swimming, resistance training, and HIIT are all promising interventions, they differ in physiological and metabolic characteristics, and their impact on diabetic lung disease and potential mechanisms may not be identical. Therefore, this study aimed to investigate and compare the effects of different exercise modalities on pulmonary inflammation and fibrosis in T2DM mice and to explore the underlying mechanisms. We hypothesized that swimming, resistance exercise, and HIIT might all improve pulmonary inflammation and fibrosis in T2DM mice by modulating oxidative stress, macrophage polarization, and EMT processes.

## 2. Materials and Methods

### 2.1. Animals

Male C57BL/6J mice (4 weeks old) were obtained from the Model Animal Research Center of Nanjing University (Nanjing, China). All animals were maintained under specific pathogen-free (SPF) conditions in the barrier facility of the Laboratory Animal Center at Shanghai University of Sport. The environment was maintained under a 12-h light/dark cycle, temperature between 21–25 °C, and relative humidity of 40–50%. Mice had unrestricted access to food and water throughout the experiment. Daily food intake was recorded, and body weight was measured every Friday. All procedures were approved by the Ethics Review Committee for Animal Experimentation of Shanghai University of Sport (Approval No. 102772019DW009).

### 2.2. T2DM Mouse Model Induction

As previously reported in our earlier studies [28,29], a T2DM model was induced by combining a high-fat diet (HFD) with a streptozotocin (STZ) injection. Following one week of acclimatization, animals were randomly assigned into two groups: a standard diet group (CON, n = 10) receiving normal chow (D12450J; 3.85 kcal/g, 10% fat, 20% protein; SYSE Ltd., Nanjing, China), and an HFD group (n = 60) fed a high-fat formulation (D12492; 5.24 kcal/g, 60% fat, 20% protein; SYSE Ltd., Nanjing, China). After 12 weeks of dietary intervention, mice in the HFD group received an intraperitoneal injection of STZ (100 mg/kg, S0130, Sigma-Aldrich, St. Louis, MO, USA), dissolved in 0.1 mmol/L sodium citrate buffer (pH 4.4), while the CON group received vehicle injections. Seven days later, fasting blood glucose (FBG), glucose tolerance, and insulin tolerance were evaluated. Mice with FBG ≥ 16.7 mmol/L were considered diabetic [30]. Successfully induced diabetic mice were then randomly divided into four groups: sedentary diabetic control (T2DM-SED, n = 9), swimming exercise (T2DM-SW, n = 9), resistance exercise (T2DM-RE, n = 9), and high-intensity interval training (T2DM-HIIT, n = 9).

### 2.3. Exercise Protocol

**Figure 1:**
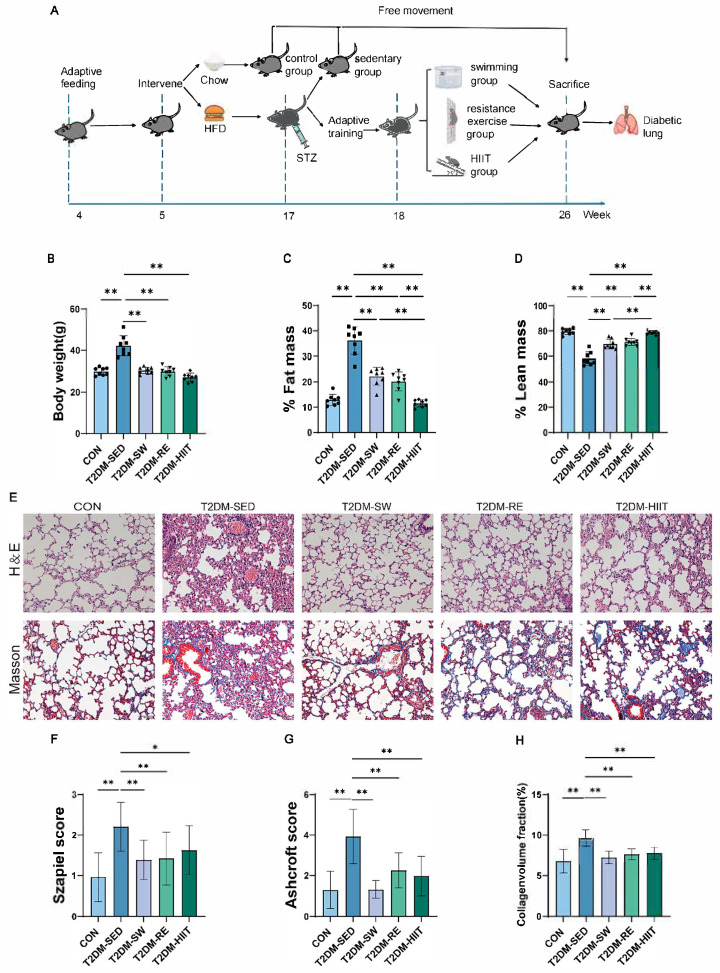
Exercise improved body composition and lung morphopathology and collagen fiber deposition in T2DM mice. (**A**) Experimental design diagram. (**B**) Body weight (n = 8 per group). (**C**) Percent fat mass (n = 8 per group). (**D**) Percent lean mass (n = 8 per group). (**E**) Representative images of H&E and Masson staining (scale bar, 50 μm). (**F**) The Szapiel score was used for the quantitative evaluation of H&E-stained lung tissue sections of mice (n = 3 per group). (**G**) The Ashcroft score was used for the quantitative evaluation of Masson-stained lung tissue sections of mice (n = 3 per group). (**H**) The quantification of positive areas of Masson staining in the mice lungs (n = 3 per group). All data are presented as the mean ± SD. * *p* < 0.05, ** *p* < 0.01.

The exercise protocols followed our previously published methods [31]. Mice in the exercise groups underwent 9 weeks of training, including 1 week of adaptation. Throughout the study, dietary conditions remained consistent: control mice were maintained on a standard diet, while diabetic mice continued to receive a high-fat diet. The experimental flow is outlined in Figure 1A.

#### 2.3.1. Swimming

The protocol for swimming exercise was designed and modified based on previously published articles [27,32]. Mice were placed in a water-filled plastic container (water temperature maintained at 35 °C) for weight-free swimming. During the adaptation week, swimming duration increased from 10 min to 60 min per day in 10 min increments. From week 2 onward, formal swimming sessions were held for 60 min per day, 5 days per week, over 8 weeks.

#### 2.3.2. Resistance Exercise

The protocol for resistance exercise was designed and modified based on previously published article [33]. Resistance exercise was performed using ladder climbing (ladder length 1 m, width 0.18 m, with 2 cm between steps; the ladder was positioned at an 85° angle to the floor). Weights were attached to the upper part of the mouse’s tail, and mice were encouraged to climb by gentle tapping. During the adaptation phase, mice climbed without weight, and loads were progressively increased until reaching 30% of body weight. From the second week onwards, mice completed four sets of ladder climbs per day (2 min rest between sets), with each set consisting of five climbs (1 min rest between climbs), 5 days per week for 8 weeks. The weight load was progressively increased from 30% to 100% of body weight over the 8 weeks: 30% in week 1; 55% in week 2; 80% in weeks 3–4; 90% in weeks 5–6; and 100% in weeks 7–8.

#### 2.3.3. High-Intensity Interval Training (HIIT)

HIIT was performed on a treadmill inclined at 25°, following the modified method [28,34]. During adaptation, mice were familiarized with the treadmill, with gradual increases in speed and slope. The training sessions consisted of a 10-min warm-up at 5 m/min, followed by 10 cycles of high-speed running for 4 min each, interspersed with 2 min passive rest intervals. Treadmill speed was progressively increased over 8 weeks—from 16 m/min to 26 m/min—with 2 m/min increments during the first 4 weeks and 1 m/min increments in the final 4 weeks.

### 2.4. Tissue Collection

After completing the exercise intervention, all animals were anesthetized with 3% isoflurane via spontaneous inhalation and subsequently euthanized by cervical dislocation. The left lung was dissected, rinsed with saline to remove residual blood, and fixed in 4% paraformaldehyde for histopathological evaluation. The right lung was immediately snap-frozen in liquid nitrogen and stored at −80 °C for later biochemical analyses.

### 2.5. Body Composition

After 8 weeks of intervention, body composition was evaluated using EchoMRI (Echo Medical Systems, Houston, TX, USA). Lean mass and fat mass were recorded, and body fat percentage was calculated as the ratio of fat mass to body weight.

### 2.6. Hematoxylin-Eosin (H&E) Staining

Lung samples fixed in 4% paraformaldehyde for 48 h were processed through graded ethanol dehydration, embedded in paraffin, and cut into 5 μm sections. Standard H&E staining procedures were applied, including 5 min hematoxylin staining, differentiation in acid alcohol, bluing under tap water, 3 min eosin staining, and mounting with neutral resin. Lung tissue morphology was observed and imaged using an OLYMPUS microscope (Olympus, Tokyo, Japan) at 200× magnification. Six random fields per section were evaluated using the Szapiel scoring system to assess lung injury [35,36].

### 2.7. Masson Staining

Lung tissue sections were prepared as described above. Masson’s trichrome staining was performed using a commercial kit (G1006, Servicebio, Wuhan, China) according to the manufacturer’s instructions. Sections were sealed with neutral resin and observed under an OLYMPUS microscope at 200× magnification. Six random fields per section were evaluated for lung fibrosis severity using the Ashcroft scoring system [35,37]. The positively stained fibrotic areas were quantified using ImageJ software 1.53a (National Institutes of Health, Bethesda, MD, USA).

### 2.8. Immunofluorescence Staining

Paraffin-embedded lung sections were deparaffinized and rehydrated through graded ethanol. Sections were incubated overnight at 4 °C with primary antibodies against CD68 (#97778, Cell Signaling Technology, Danvers, MA, USA, 1:250), CD11c (#97585, Cell Signaling Technology, USA, 1:250), CD206 (#24595, Cell Signaling Technology, USA, 1:250), Fibronectin (sc-8422, Santa Cruz Biotechnology, Dallas, TX, USA, 1:250), α-SMA (GB111364, Servicebio, China, 1:250), and E-cadherin (GB12083, Servicebio, China, 1:250). After washing with PBS, sections were incubated with fluorophore-conjugated secondary antibodies at room temperature for 90 min. Nuclear counterstaining was performed with DAPI, and images were obtained using a Zeiss LSM 700 confocal microscope (Carl Zeiss Microscopy, Jena, Germany). Six random fields per section were analyzed, and positive staining areas were quantified using ImageJ software.

### 2.9. RNA Extraction and Gene Expression Analysis

Total RNA was isolated from lung tissue using Total RNA Isolation Reagent (BS258A, Labgic, Beijing, China). The purity and concentration of RNA were evaluated by measuring absorbance at 260 and 280 nm using a NanoDrop™ One spectrophotometer (Thermo Scientific, Waltham, MA, USA). cDNA was synthesized using a cDNA Synthesis Kit (AG11706, Accurate Biotechnology [Hunan] Co., Ltd., Changsha, China). Real-time quantitative PCR (RT-qPCR) was performed using the SYBR Green Premix Pro Taq HS qPCR Kit (Rox Plus) (AG11701, Accurate Biotechnology [Hunan] Co., Ltd., Changsha, China). Relative gene expression was calculated using the 2^−ΔΔCt^ method, with β-actin as the housekeeping gene. The primer sequences used in this study are listed in Table 1.

### 2.10. Western Blotting Analysis

Lung tissues were lysed using RIPA buffer (P0013B, Beyotime, Shanghai, China), and protein concentrations were measured with a BCA assay kit (P0010, Beyotime, Shanghai, China) following the manufacturer’s protocol. Protein samples (40 μg per lane) were denatured by boiling and separated by electrophoresis on 10% SDS-PAGE gels, followed by transfer to 0.45 μm PVDF membranes (IPVH00010, Millipore, Bedford, MA, USA). The membranes were blocked with 5% non-fat milk or BSA at room temperature for 90 min. Based on molecular weight markers, membranes were cut into strips and incubated overnight at 4 °C with primary antibodies: SOD2 (sc-137254, Santa Cruz Biotechnology, USA, 1:1000), NF-κBp65 (#8242, Cell Signaling Technology, USA, 1:1000), TGF-β1 (sc-52893, Santa Cruz Biotechnology, USA, 1:1000), phospho-Smad2 (#3108, Cell Signaling Technology, USA, 1:1000), phospho-Smad3 (#9520, Cell Signaling Technology, USA, 1:1000), Smad4 (sc-7966, Santa Cruz Biotechnology, USA, 1:1000), and α-tubulin (66009-1-Ig, Proteintech, Wuhan, China, 1:10,000). The next day, membranes were washed with TBST and incubated with HRP-conjugated secondary antibodies at room temperature for 90 min. After additional washing with TBST, bands were visualized using enhanced chemiluminescence (ECL) reagents and imaged with a Tanon gel imaging system. Semi-quantitative analysis of protein expression was performed using ImageJ software, with the expression level represented by the ratio of the target protein band intensity to the loading control.

### 2.11. Statistical Analysis

All data are presented as mean ± standard deviation (SD). Statistical analysis was conducted using SPSS 26.0 (IBM, Armonk, NY, USA), and graphs were generated using GraphPad Prism 9.0. Comparisons among groups were performed using one-way ANOVA followed by Bonferroni’s post hoc test. A *p*-value < 0.05 was considered statistically significant.

## 3. Results

### 3.1. Exercise Reduced Body Weight and Body Fat Percentage in T2DM Mice

We first assessed changes in body weight and body fat percentage in T2DM mice induced by HFD combined with a single STZ injection, as well as the effects of exercise interventions. MRI analysis showed that, compared to the CON group, the T2DM-SED group exhibited significantly increased body weight and body fat percentage, along with a significant decrease in lean mass content (Figure 1B–D). After 8 weeks of exercise intervention, all exercise groups demonstrated significantly reduced body weight and body fat percentage, as well as increased lean mass content compared to the T2DM-SED group (Figure 1B–D). Notably, the T2DM-HIIT group showed the lowest body fat percentage and the highest lean mass percentage, indicating that HIIT was the most effective intervention for fat reduction.

### 3.2. Exercise Improved Pulmonary Morphological Damage and Fibrosis in T2DM Mice

Alveolar structural damage, inflammatory cell infiltration, and extracellular matrix deposition in the lung interstitium are hallmark pathological features of diabetic lung disease. To assess the effects of three different types of exercise on pulmonary morphology, inflammation, and collagen fiber deposition in T2DM mice, H&E and Masson’s staining were performed on lung tissues. Histological changes were semi-quantitatively evaluated using the Szapiel and Ashcroft scoring systems. H&E staining showed that, compared to the CON group, the T2DM-SED group exhibited significant alveolar destruction, thickening of alveolar septa and vascular basement membranes, airway epithelial hyperplasia, and marked infiltration of inflammatory cells (Figure 1E), with significantly increased Szapiel scores (Figure 1F). Masson staining revealed substantial increases in fibrotic areas, honeycomb-like structures, and thickening of the interstitium, airway walls, and vascular walls due to collagen fiber deposition in the T2DM-SED group (Figure 1E,H), with significantly elevated Ashcroft scores (Figure 1G). These pathological changes were markedly improved in all three exercise groups compared to the T2DM-SED group, with significant reductions in both Szapiel and Ashcroft scores (Figure 1F,G). Importantly, among the three exercise groups, the T2DM-SW group demonstrated the most pronounced reduction in both scores, indicating the strongest protective effect.

### 3.3. Exercise Attenuated Oxidative Stress in the Lungs of T2DM Mice

**Figure 2:**
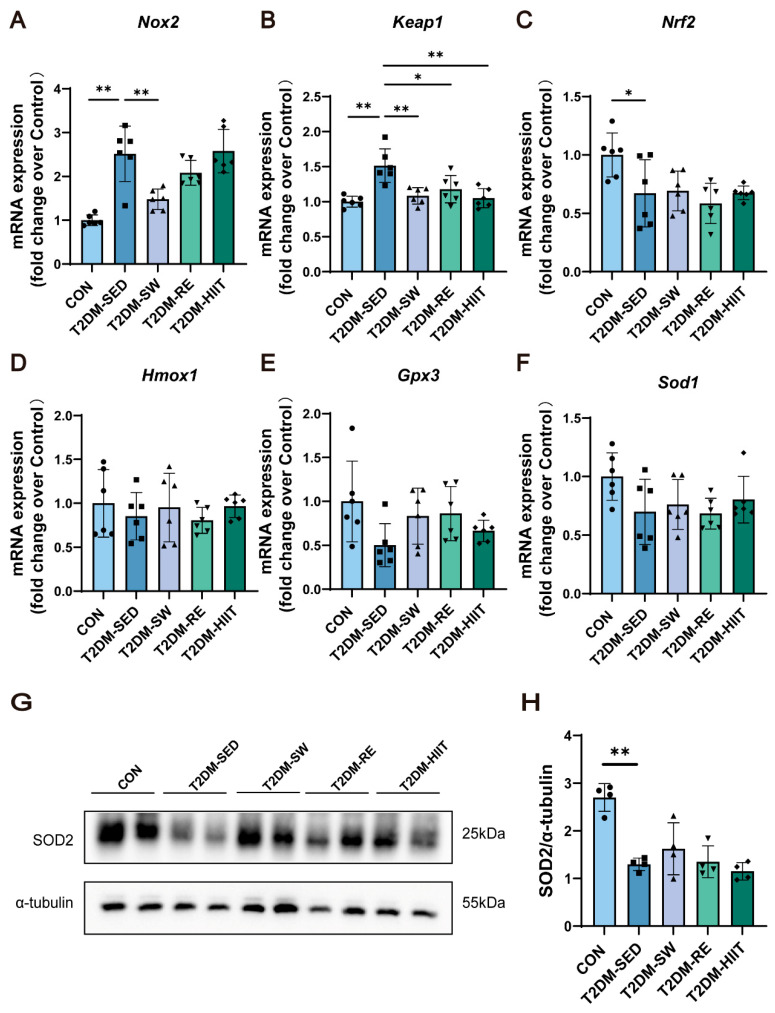
Exercise improves oxidative stress in lung tissue of T2DM mice. (**A**–**F**) The mRNA expression levels of *Nox2*, *Keap1*, *Nrf2*, *Hmox1*, *Gpx3*, *and Sod1* (n = 6 mice per group). (**G**,**H**) SOD2 protein expression (n = 4 mice per group). All data are presented as the mean ± SD. * *p* < 0.05, ** *p* < 0.01.

T2DM mice exhibited significant oxidative stress injury. To assess this, we measured mRNA and protein expression levels of oxidative stress-related and antioxidant molecules in lung tissues. Compared to the CON group, the T2DM-SED group showed significantly elevated mRNA expression of the oxidative stress marker *Nox2* (Figure 2A), along with suppression of the *Keap1/Nrf2/Hmox1* antioxidant pathway and reduced mRNA expression of antioxidant enzymes *Gpx3* and *Sod1* (Figure 2B–F). All three exercise interventions significantly reduced *Nox2* and *Keap1* expression, activated the *Keap1/Nrf2/Hmox1* signaling pathway, and increased the expression of *Gpx3* and *Sod1* (Figure 2A–F). Notably, swimming resulted in the most significant reduction in *Nox2* gene expression (Figure 2A,B). Furthermore, we detected the protein expression of SOD2, a key antioxidant enzyme in lung tissue. Compared to the CON group, the T2DM-SED group exhibited significantly decreased SOD2 protein levels, while all exercise modalities increased SOD2 expression without significant differences between them (Figure 2G,H). These findings suggest that all three types of exercise partially alleviate oxidative stress in the lungs of T2DM mice.

### 3.4. Exercise Suppressed the Expression of Inflammatory Cytokines in the Lungs of T2DM Mice

**Figure 3:**
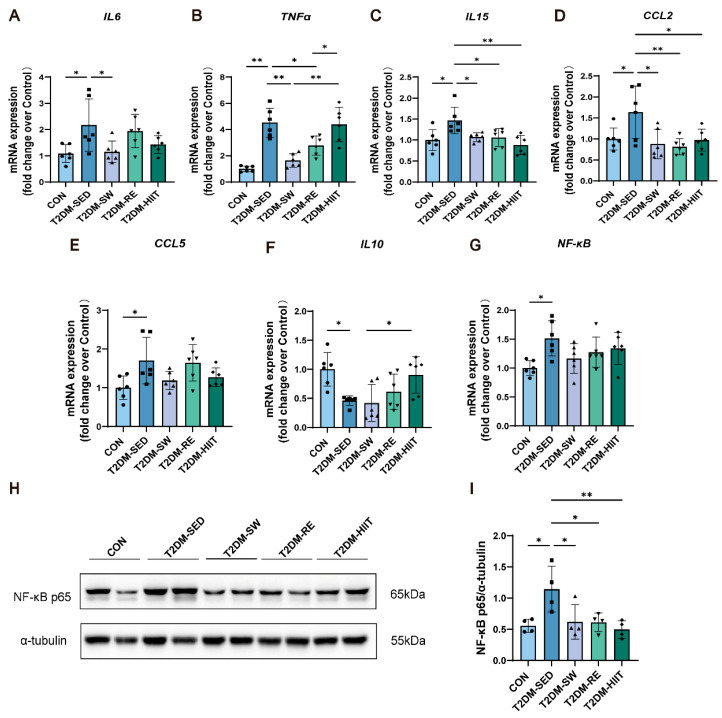
Exercise improved lung tissue inflammation in T2MD mice. (**A**–**G**) The mRNA expression levels of *IL6*, *TNFα*, *IL15*, *CCL2*, *CCL5*, *IL10*, and *NF-κB* (n = 6 mice per group). (**H**,**I**) NF-κB p65 protein expression (n = 4 mice per group). All data are presented as the mean ± SD. * *p* < 0.05, ** *p* < 0.01.

Pulmonary inflammatory cell infiltration and chronic inflammation are key pathological features of diabetic lung disease [16,38,39]. We measured the expression of inflammatory cytokines in lung tissue. Compared to the CON group, the T2DM-SED group exhibited significantly elevated mRNA expression of pro-inflammatory cytokines *IL-6*, *TNF-α*, and *IL-15* (Figure 3A–C), as well as chemokines *CCL2* and *CCL5* (Figure 3D,E). Conversely, the mRNA expression of the anti-inflammatory cytokine *IL-10* was significantly reduced (Figure 3F). All three types of exercise intervention significantly reduced the mRNA expression of pro-inflammatory cytokines and chemokines (Figure 3A–E), while increasing *IL-10* expression (Figure 3F). The nuclear factor kappa-B (NF-κB) pathway is closely associated with inflammation and immune regulation and has long been considered a therapeutic target for inflammatory diseases. Previous studies have confirmed that activation of NF-κB and its downstream effectors plays a critical role in T2DM-related lung injury [26]. We therefore assessed NF-κB p65 expression at both mRNA and protein levels. Compared to the CON group, the T2DM-SED group showed significantly elevated *NF-κB* mRNA expression, which was reduced by all three exercise interventions, although without statistical significance (Figure 3G). At the protein level, NF-κB p65 expression was significantly increased in the T2DM-SED group, while all three exercise interventions markedly reduced NF-κB p65 protein levels (Figure 3H,I). These results indicate that lung inflammatory responses are markedly enhanced in T2DM mice induced by 12 weeks of HFD combined with a single STZ injection, and that 8 weeks of swimming, resistance exercise, and HIIT can significantly suppress this inflammatory response.

### 3.5. Exercise Reduced Macrophage Infiltration, Inhibited Pro-Inflammatory Macrophage Polarization, and Promoted Anti-Inflammatory Polarization in the Lungs of T2DM Mice

**Figure 4:**
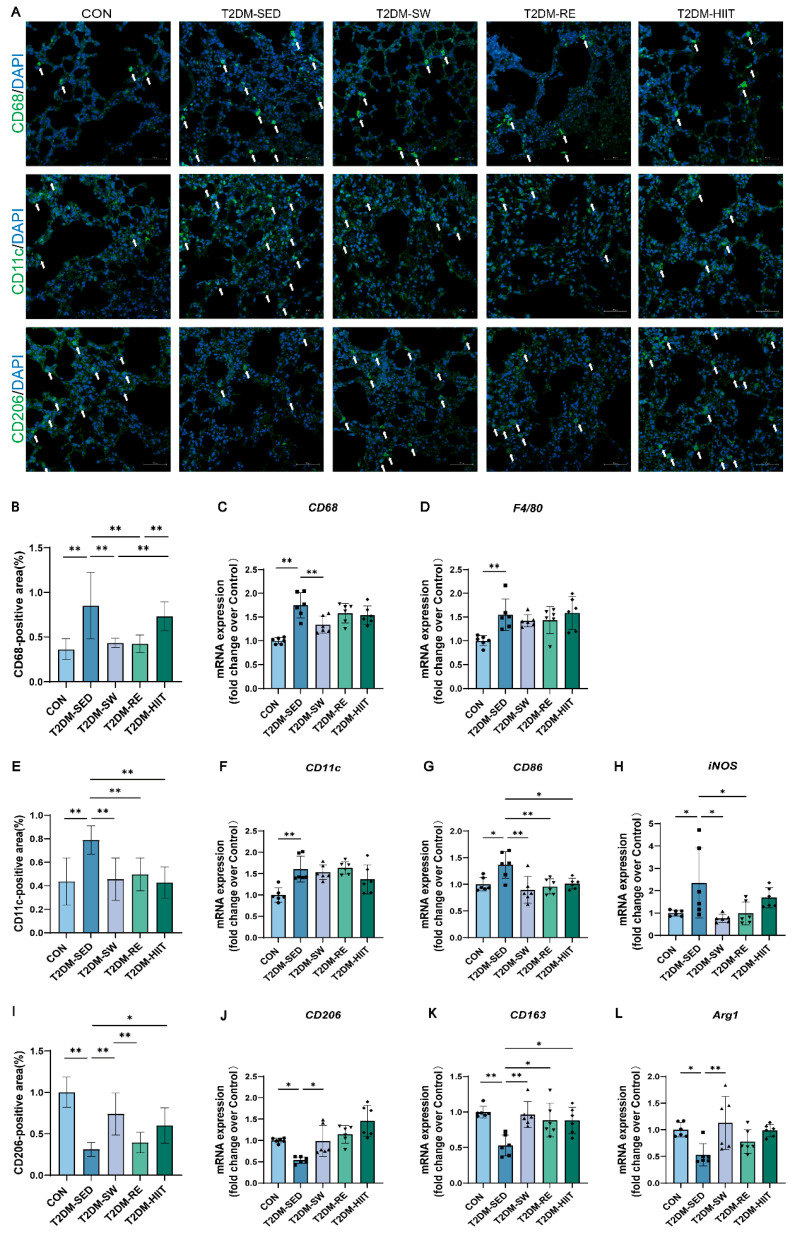
Exercise inhibited macrophage infiltration, inhibited pro-inflammatory macrophage polarization, and promoted anti-inflammatory polarization in lung tissue of T2DM mice. (**A**) Representative images of CD68, CD11c, and CD206 immunofluorescence staining (scale bar, 50 μm). The white arrowheads indicate the localization of CD68, CD11c, and CD206. (**B**,**E,I**) Quantification of CD68-positive, CD11c-positive, and CD206-positive area (n = 3 mice per group). (**C**,**D**,**F**–**H**,**J**–**L**) The mRNA expression levels of *CD68*, *F4/80*, *CD11c*, *CD86*, *iNOS*, *CD206*, *CD163*, and *Arg1* (n = 6 mice per group). All data are presented as the mean ± SD. * *p* < 0.05, ** *p* < 0.01.

Macrophages play a key role in lung tissue injury and repair. We first examined the expression of total macrophage markers to assess infiltration and activation in the lung tissue. Immunofluorescence analysis showed that, compared to the CON group, the number and density of macrophages in lung tissue were markedly increased in the T2DM-SED group, while all three types of exercise reduced macrophage numbers and infiltration density (Figure 4A,B). RT-qPCR results further demonstrated that the mRNA expression levels of general macrophage markers *CD68* and *F4/80* were significantly elevated in the T2DM-SED group compared to the CON group, while all three exercise interventions showed a decreasing trend, with swimming exercise causing a significant reduction in *CD68* mRNA expression (Figure 4C,D). These results indicate that exercise training effectively reduces macrophage infiltration in the lungs of T2DM mice. Alveolar macrophages display considerable heterogeneity in phenotype and function. M1-type macrophages promote inflammation by releasing pro-inflammatory cytokines, while M2-type macrophages are associated with anti-inflammatory effects and tissue repair. To investigate macrophage polarization in the lungs under T2DM conditions, we assessed M1 and M2 macrophage marker expression by immunofluorescence and RT-qPCR. Immunofluorescence results showed that the number and density of CD11c-positive M1 macrophages were markedly increased in the T2DM-SED group, while CD206-positive M2 macrophages were significantly reduced compared to the CON group (Figure 4A,B,F). All three exercise interventions decreased M1 macrophage numbers and increased M2 macrophage counts (Figure 4A,E,I). RT-qPCR analysis further confirmed that the mRNA expression levels of M1 markers (*CD11c*, *CD86*, and *iNOS*) were significantly elevated in the T2DM-SED group (Figure 4F–H), whereas M2 markers (*CD206*, *CD163*, and *Arg1*) were significantly downregulated (Figure 4J–L). All three exercise interventions reduced M1 marker expression and upregulated M2 marker expression in lung tissue (Figure 4F–H,J–L). These findings demonstrate that exercise suppresses pro-inflammatory M1 polarization and promotes anti-inflammatory M2 polarization of macrophages in the lungs of T2DM mice.

### 3.6. Exercise Ameliorated Pulmonary Fibrosis in T2DM Mice

**Figure 5:**
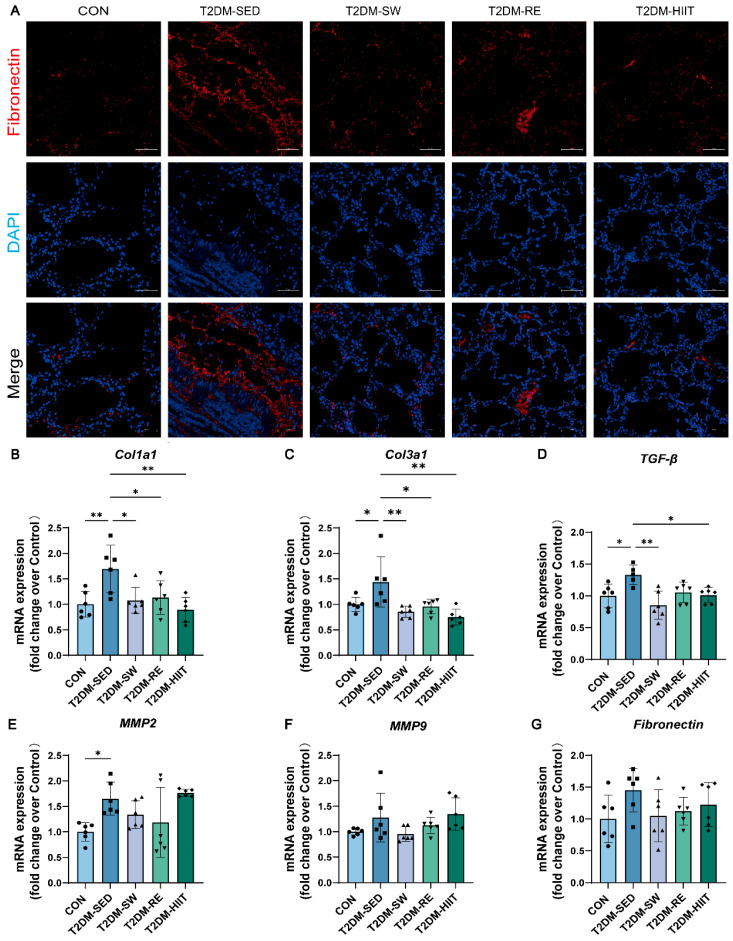
Exercise improved pulmonary fibrosis in T2DM mice. (**A**) Representative images of Fibronectin immunofluorescence staining (scale bar, 50 μm). (**B**–**G**) The mRNA expression levels of *Col1a1*, *Col3a1*, *TGF-β*, *MMP2*, *MMP9*, and *Fibronectin* (n = 6 mice per group). All data are presented as the mean ± SD. * *p* < 0.05, ** *p* < 0.01.

To further investigate the effects of three different types of exercise on pulmonary fibrosis in T2DM mice, we performed immunofluorescence staining for Fibronectin to assess the content of extracellular matrix (ECM) components. Additionally, the mRNA expression levels of other fibrosis-related markers were measured. Immunofluorescence staining revealed that, compared to the CON group, the T2DM-SED group exhibited a higher level of Fibronectin in the lung tissue, which was significantly downregulated after exercise (Figure 5A). RT-qPCR results indicated that, compared to the CON group, the mRNA expressions of *Col1a1*, *Col3a1*, *TGF-β*, *MMP2*, *MMP9*, and *Fibronectin* were significantly elevated in the T2DM-SED group (Figure 5B–G). All three exercise types significantly reduced the mRNA expression of *Col1a1*, *Col3a1*, *TGF-β*, *MMP2*, *MMP9*, and *Fibronectin* (Figure 5B–G). These findings suggest that exercise alleviates T2DM-induced pulmonary fibrosis.

### 3.7. Exercise Suppressed EMT in the Lungs of T2DM Mice

**Figure 6:**
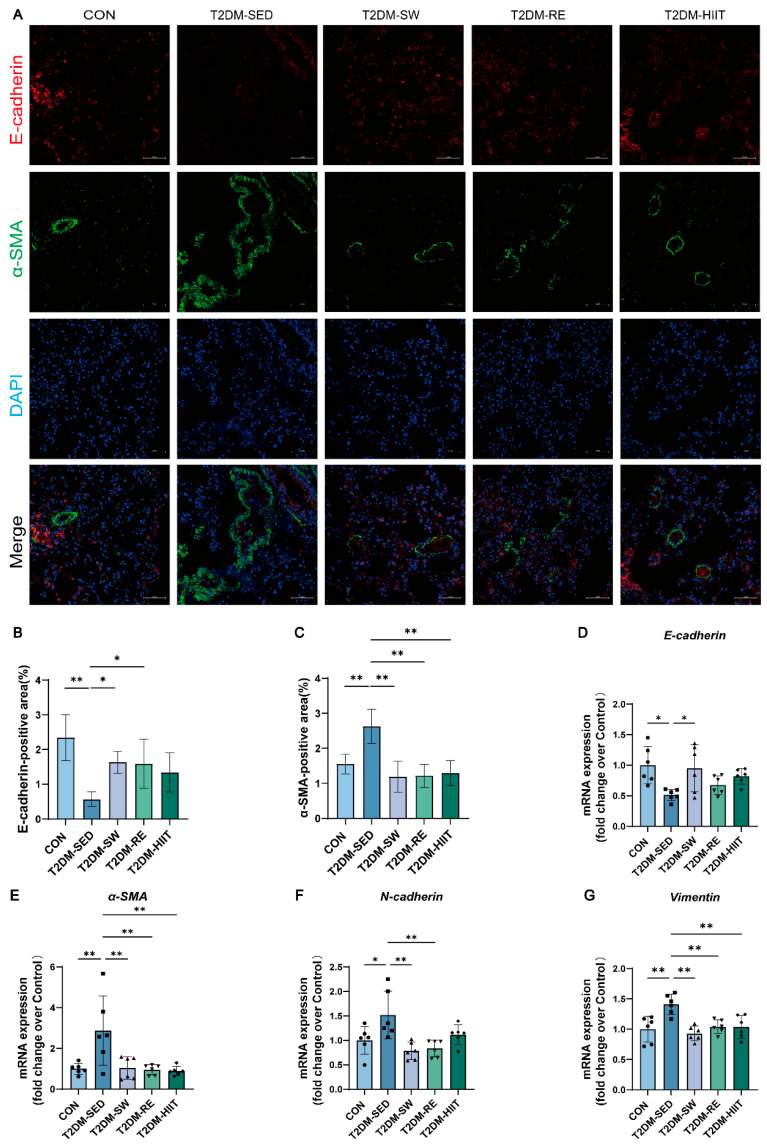
Exercise inhibited EMT in lung tissue of T2DM mice. (**A**) Representative images of E-cadherin and α-SMA immunofluorescence staining (scale bar, 50 μm). (**B**,**C**) Quantification of E-cadherin-positive and α-SMA-positive area (n = 3 mice per group). (**D**–**G**) The mRNA expression levels of *E-cadherin*, *α-SMA*, *N-cadherin*, and *Vimentin* (n = 6 mice per group). All data are presented as the mean ± SD. * *p* < 0.05, ** *p* < 0.01.

EMT is a critical pathological process in tissue fibrosis progression. To evaluate the EMT activity in lung tissue under T2DM conditions, we performed dual immunofluorescence staining for the epithelial marker E-cadherin and the mesenchymal marker α-smooth muscle actin (α-SMA), along with RT-qPCR to measure the expression of EMT-related genes. The results of immunofluorescence staining showed that, compared to the CON group, the T2DM-SED group exhibited a significant reduction in epithelial cell numbers and an increase in mesenchymal cells, with all three exercise types improving this condition (Figure 6A–C). RT-qPCR analysis revealed that, compared to the CON group, the mRNA expression of mesenchymal markers *α-SMA*, *N-cadherin*, and *Vimentin* was significantly upregulated in the T2DM-SED group, and all three exercise types significantly reduced their expression (Figure 6E–G). Conversely, compared to the CON group, the mRNA expression of the epithelial marker *E-cadherin* was significantly decreased in the T2DM-SED group, and all three exercise types elevated its expression, with swimming showing a statistically significant difference (Figure 6D).

### 3.8. Exercise Inhibited TGF-β1/Smad Signaling Pathway Activation in the Lungs of T2DM Mice

**Figure 7:**
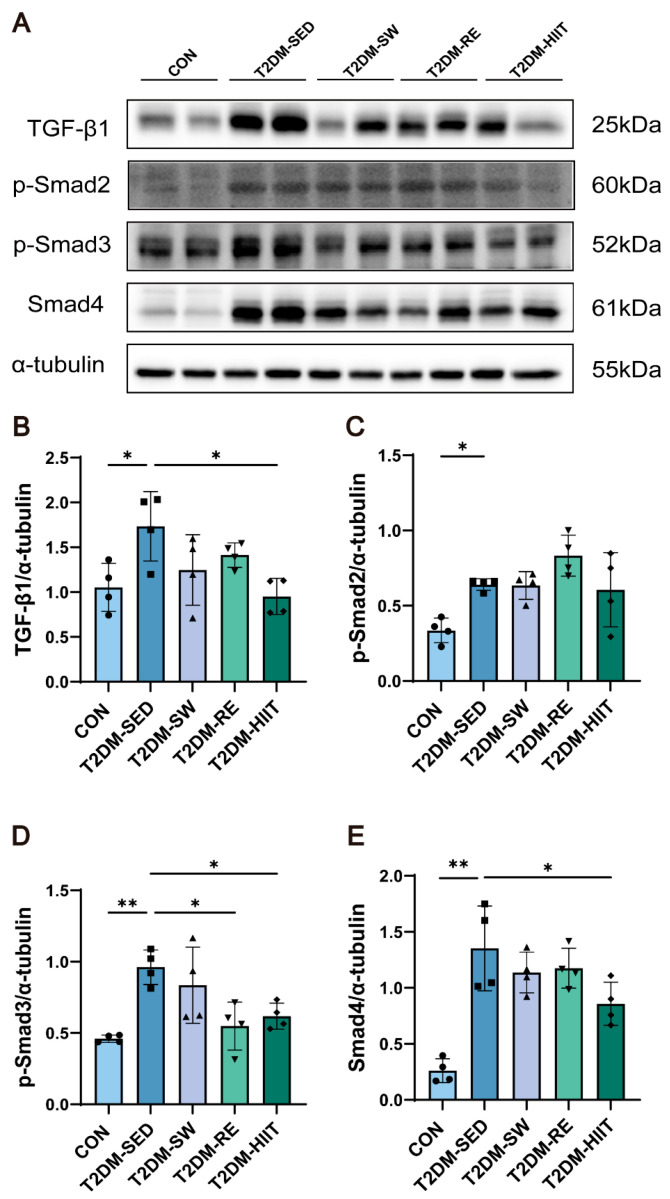
Exercise inhibited the activation of TGF-β1/Smad signaling pathway in lung tissue of T2DM mice. (**A**–**E**) TGF-β1, p-Smad2, p-Smad3, and Smad4 protein expression (n = 4 mice per group). All data are presented as the mean ± SD. * *p* < 0.05, ** *p* < 0.01.

The TGF-β1/Smad signaling pathway plays a crucial role in regulating EMT and fibrosis progression. Previous studies have shown that TGF-β1/Smad signaling activity is increased in the lung tissue of diabetic rats [19]. To verify the activation of the TGF-β1/Smad signaling pathway in the lungs of T2DM mice and the impact of exercise, we measured the expression of TGF-β1/Smad pathway-related proteins. Compared with the CON group, the protein levels of TGF-β1, p-Smad2, p-Smad3, and Smad4 in the T2DM-SED group were significantly elevated. All three types of exercise interventions demonstrated a tendency to reduce the expression levels of proteins involved in the TGF-β1/Smad signaling pathway to varying extents (Figure 7A–E). Notably, HIIT significantly down-regulated the protein expressions of TGF-β1, p-Smad3, and Smad4, suggesting that HIIT exerts a relatively superior inhibitory effect on the activation of the TGF-β1/Smad signaling pathway.

## 4. Discussion

Disturbances in glucose and lipid metabolism are core pathological features of T2DM and are closely associated with impaired lung function and increased susceptibility to pulmonary diseases [40,41]. Exercise, as a key therapeutic strategy for diabetes, has been demonstrated to significantly improve systemic metabolic status and reduce various complications [42]. Both previous studies and our current findings indicate that moderate aerobic exercise, resistance training, and HIIT can reduce body weight and fat percentage while increasing lean mass ratio, suggesting that these exercise modalities effectively alleviate glucose and lipid metabolism disorders in T2DM mice. The potential mechanisms include improvement of mitochondrial dynamics, enhancement of mitochondrial biogenesis [33,43], promotion of white adipose tissue browning [34], activation of the AMP-activated protein kinase (AMPK) signaling pathway [44], and improvement of insulin sensitivity [28]. Consistent with these findings, our study showed that all three types of exercise reduced body weight and fat percentage while increasing lean body mass, indicating improvements in metabolic status in T2DM mice. Given that obesity is a major feature and contributing factor in T2DM, and has been reported to independently induce chronic pulmonary inflammation and fibrosis [45,46], the improvement in lung function observed in the exercised mice may be partly attributed to weight loss.

In hyperglycemic states, excessive accumulation of advanced glycation end products (AGEs) induces overproduction of reactive oxygen species (ROS) and inflammatory factors, resulting in damage to cellular DNA, proteins, and lipids, thereby activating multiple signaling pathways and accelerating the development of diabetic complications [47,48]. Persistent elevation of ROS levels, impairment of antioxidant defenses, and activation of nicotinamide adenine dinucleotide phosphate oxidase further exacerbate oxidative stress and pulmonary injury in diabetes [49,50,51]. The body’s endogenous antioxidant defense system, notably the Keap1/Nrf2/Hmox1 pathway, plays a key regulatory role by inducing the expression of antioxidant enzymes such as SOD and Gpx3 [52,53]. In our study, excessive activation of Nox2 and downregulation of antioxidant enzymes were observed in lung tissues of T2DM mice, indicating significant oxidative stress injury. Exercise interventions effectively suppressed Nox2 expression and enhanced activation of the Nrf2 pathway and downstream antioxidant enzymes, suggesting that all three exercise modalities contributed to attenuating oxidative stress and strengthening pulmonary antioxidant defenses, consistent with previous research findings [26,46,54,55].

Systemic chronic low-grade inflammation is a key pathogenic mechanism underlying T2DM and its complications [56]. The lungs, with their dense alveolar-capillary network and abundant connective tissue, are particularly vulnerable to inflammatory injury triggered by the accumulation of AGEs [57]. Our study demonstrated that T2DM induces significant pathological changes in lung tissue, including inflammatory cell infiltration, alveolar septal destruction, collapse, interstitial proliferation, and increased vascular permeability. Different types of exercise interventions markedly improved these pathological changes and reduced Szapiel scores, with swimming exercise showing the most pronounced protective effect, consistent with previous findings [25,26,27]. Notably, this study is the first to report the anti-inflammatory effects of resistance exercise in alleviating pulmonary inflammation in T2DM mice. Increased expression of pro-inflammatory cytokines, such as IL-1β, IL-6, and TNF-α, and chemokines like CCL2 and CCL5, are hallmarks of inflammatory responses [58]. Downregulation of anti-inflammatory cytokines, such as IL-10, may lead to uncontrolled inflammation and exacerbate tissue injury. Previous studies have shown elevated expression of IL-1β, IL-6, TNF-α, and CCL2 in the lungs of T2DM models, accompanied by decreased IL-4 and IL-10 expression, resulting in enhanced inflammatory cell infiltration and lung tissue damage [16,46]. Suppression of inflammation has been shown to effectively alleviate diabetic lung injury [16]. The NF-κB signaling pathway is central to regulating inflammatory responses and can promote the expression of multiple pro-inflammatory cytokines when activated [59]. Increased nuclear NF-κB levels in the lung tissues of diabetic rats have been reported, and its inhibition significantly reduces inflammation and tissue damage [54,60]. The anti-inflammatory effect of exercise is also well documented [61]. Athari et al. [26] reported that voluntary exercise significantly reduced the expression level of NF-κB p65 protein in lung tissue of T2DM rats and improved lung inflammation. Consistent with these findings, our study showed that NF-κB mRNA and protein levels were elevated in the lungs of T2DM mice, and exercise intervention effectively reduced NF-κB expression, with HIIT showing the strongest suppression, possibly due to the more potent physiological stress response induced by its high-intensity intervals [62]. In summary, our findings further confirm that exercise, particularly swimming and HIIT, exerts significant anti-inflammatory effects in the lungs of T2DM mice.

Alveolar macrophages are the most abundant immune cells in the lungs and play critical roles in maintaining pulmonary immune homeostasis, regulating inflammation, and participating in tissue repair and fibrosis [63]. As part of the innate immune system, macrophage activation is considered an early response to lung injury [64]. Their excessive activation and polarization imbalance are key mechanisms in chronic pulmonary inflammation and fibrosis. High glucose conditions have been shown to stimulate the synthesis of hyaluronic acid in airway smooth-muscle extracellular matrices, promoting monocyte-macrophage recruitment and leading to chronic inflammation and fibrosis, thereby aggravating diabetic lung injury [65]. In this study, we found significantly increased expression of macrophage markers CD68 and F4/80 in the lung tissue of T2DM mice, indicating robust macrophage infiltration and activation. Activated macrophages likely release inflammatory cytokines and chemokines, further recruiting immune cells and exacerbating pulmonary inflammation. Moreover, we observed significant increases in M1 macrophage markers (CD11c, CD86, iNOS) and decreases in M2 macrophage markers (CD206, CD163, Arg1), indicating a predominance of pro-inflammatory M1 polarization and suppression of anti-inflammatory M2 polarization, consistent with findings by Aisanjiang et al. [39]. Our previous work has demonstrated that HIIT promotes anti-inflammatory macrophage polarization in the liver and white adipose tissue of T2DM mice [28,34]. The current study further confirms that all three exercise modalities reduce macrophage infiltration and activation, promote M1-to-M2 polarization shifts, and enhance pulmonary anti-inflammatory and reparative capacity, thereby effectively alleviating lung inflammation induced by T2DM.

Pulmonary fibrosis is a key pathological feature of diabetic lung injury, characterized by excessive proliferation of connective tissue and loss of parenchymal cells, ultimately resulting in impaired lung function [66,67]. Previous studies have demonstrated that STZ-induced diabetic rats exhibit increased lung volume and weight, elevated collagen deposition, and reduced connective tissue turnover [68]. Consistent with these findings, our study observed significant interstitial collagen accumulation, thickening of the tracheal and vascular walls, and upregulation of fibrosis-related genes in the lung tissue of T2DM mice, suggesting that chronic hyperglycemia and metabolic disturbances promote ECM deposition and pulmonary fibrosis. All three exercise interventions partially reversed these changes and downregulated fibrotic markers, with swimming and HIIT showing the most pronounced effects, in line with existing research [25,69]. Importantly, this study is the first to report that resistance exercise also contributes to improving pulmonary fibrosis in T2DM mice. EMT is a critical process in the progression of fibrosis, characterized by the loss of epithelial characteristics and acquisition of mesenchymal features, along with alterations in marker expression. Hyperglycemia and inflammatory environments can promote EMT in lung tissue, leading to tissue remodeling and organ dysfunction [18,70]. Our results showed decreased expression of the epithelial marker E-cadherin and increased expression of mesenchymal markers α-SMA, N-cadherin, and Vimentin in the lungs of T2DM mice, indicating EMT activation. All three exercise modalities effectively reversed this process, suggesting that exercise can inhibit EMT progression. In addition to EMT, endothelial-mesenchymal transition (EndMT) also plays an important role in diabetic pulmonary fibrosis. A recent study reported that pulmonary fibrosis in T2DM patients predominantly accumulates around pulmonary vasculature [30], which is consistent with our observations. Further evidence suggests that both T2DM patients and STZ-induced diabetic mice exhibit enhanced EndMT, and pharmacological inhibition of EndMT can significantly alleviate diabetic pulmonary fibrosis [30]. This finding highlights a novel mechanism in diabetic lung fibrosis, and future studies could explore whether exercise exerts protective effects by regulating EndMT.

The TGF-β1/Smad signaling pathway is a central regulator of EMT and fibrosis [71,72,73]. Activation of TGF-β1 promotes the phosphorylation of Smad2/3 and the formation of a complex with Smad4, thereby facilitating fibroblast proliferation, differentiation into myofibroblasts, and excessive ECM deposition [74]. Previous studies have shown significant activation of the TGF-β1/Smad signaling pathway in the lungs of diabetic rats [19,71]. Therefore, inhibition of this pathway represents a promising therapeutic strategy for diabetic lung complications. Our findings confirmed that TGF-β1, p-Smad2, p-Smad3, and Smad4 expression were significantly upregulated in the lungs of T2DM mice, while exercise intervention effectively suppressed these changes, with HIIT showing the most pronounced inhibitory effect. This study is the first to demonstrate that exercise can alleviate pulmonary fibrosis and EMT progression in T2DM mice by modulating the TGF-β1/Smad pathway.

Previous studies have demonstrated that exercise can ameliorate diabetic lung injury. For example, swimming has been reported to attenuate pulmonary inflammation and apoptosis in T1DM mice [27]; voluntary exercise alleviated pulmonary inflammation in T2DM rats by modulating NF-κB and Nrf2 signaling pathways [26]; and HIIT was shown to mitigate diabetic lung injury by regulating inflammation, oxidative stress, and apoptosis [25]. However, these studies did not compare different exercise modalities, and currently, no research has examined the effects of resistance training on pulmonary complications in T2DM models. Our study is the first to directly compare the effects of swimming, resistance training, and HIIT in a T2DM lung model. In addition, we observed that different exercise modalities exerted beneficial effects on weight loss and improvement of pulmonary inflammation and fibrosis in T2DM mice, with distinct differences in efficacy. Specifically, the HIIT group exhibited the lowest body fat percentage and highest lean mass ratio, indicating superior weight-loss effects. In terms of inflammation, swimming was more effective than resistance training and HIIT in reducing pro-inflammatory cytokines, inhibiting macrophage infiltration and M1 polarization, and promoting M2 polarization. Furthermore, swimming most effectively restored alveolar architecture and reduced alveolar septal thickening and inflammatory infiltration, resulting in the lowest Szapiel scores. Regarding pulmonary fibrosis, both swimming and HIIT significantly reduced collagen deposition, lowered Ashcroft scores and collagen area, and downregulated fibrotic markers. These novel comparative findings suggest that while exercise generally exerts beneficial effects, the extent of protection may be modality-dependent. Mechanistically, all three exercise types inhibited macrophage infiltration and pro-inflammatory polarization while promoting anti-inflammatory polarization and suppressing NF-κB expression. Furthermore, they attenuated TGF-β1/Smad signaling activation and reversed EMT progression. Notably, swimming most effectively regulated EMT-related markers, whereas HIIT exerted the strongest inhibitory effect on the TGF-β1/Smad pathway. To our knowledge, this is the first study to report that exercise regulates macrophage polarization in the lungs of T2DM mice and suppresses fibrosis and EMT progression via modulation of TGF-β1/Smad signaling.

Different exercise modalities may exert distinct effects due to their divergent metabolic characteristics. A recent meta-analysis demonstrated that aerobic exercise (such as swimming) was more effective than resistance training in reducing systemic inflammatory markers, including CRP and IL-6 [75], which may help explain the particularly strong anti-inflammatory effects observed in our swimming group. In addition, previous studies have consistently confirmed that swimming uniquely enhances respiratory muscle strength, improves lung tissue elasticity, and increases pulmonary compliance—factors that contribute to better lung capacity and ventilatory efficiency, making it a particularly effective training modality for improving pulmonary function [76,77,78,79]. Such direct improvements in lung function could also contribute to the amelioration of pulmonary inflammation and fibrosis in T2DM mice. NF-κB and TGF-β1/Smad pathways are key signaling cascades mediating inflammation and fibrosis, respectively. Our results showed that HIIT exerted a stronger inhibitory effect on both pathways, potentially due to its higher stress and recovery demands, which may induce broader signal remodeling [62]. Previous studies have shown that HIIT significantly suppresses NF-κB expression in the kidney of female rats [80] and attenuates TGF-β1/Smad-mediated fibrosis in diabetic liver [81]. Wang et al. [46] also reported that aerobic exercise reduces pulmonary TGF-β1 levels in obese mice. Moreover, HIIT may more effectively stimulate pulmonary endothelial cells. Brown et al. [82] found that HIIT improved cardiopulmonary function in rats with pulmonary hypertension more effectively than continuous training, potentially by enhancing pulmonary eNOS expression. Additionally, our previous studies demonstrated that HIIT more robustly modulates adipose tissue function, reduces adipocyte hypertrophy and inflammation, enhances mitochondrial function, promotes fatty acid oxidation, and induces browning of white adipose tissue [31]. These benefits may further alleviate lipid toxicity-induced fibrosis in both adipose and lung tissues under T2DM conditions. In contrast, resistance training appears to provide more benefits via enhancing skeletal muscle insulin sensitivity but has limited effects on circulating cytokines [83,84]. In summary, these differences in exercise-induced molecular signaling likely underlie the mode-specific effects we observed. It is worth noting that the observed differences among exercise types may partly reflect disparities in exercise intensity or volume. Although the protocols used were based on validated models, total energy expenditure was not directly measured or matched between groups. Thus, we cannot fully attribute the outcomes solely to exercise modality. Future studies incorporating matched energy expenditure or controlled workload designs will be helpful to isolate modality-specific effects.

This study has several limitations. First, only three types of exercise interventions (swimming, resistance training, and HIIT) were examined, and the intensity and frequency settings for each exercise type were relatively fixed. We did not perform a systematic comparison of different intensities, frequencies, or durations of exercise, and the study lacked a clear investigation of the “dose–response” relationship. Future studies employing metabolic workload-matched interventions would be critical to distinguish between the effects of exercise type and intensity more precisely. Second, the mechanistic exploration in this study was relatively limited. Although we preliminarily examined the roles of the TGF-β1/Smad and NF-κB signaling pathways in pulmonary inflammation and fibrosis, other potential mechanisms such as EndMT and mitochondrial homeostasis were not further validated. Regarding the role of macrophages, although our findings suggest a potential involvement of macrophage polarization in the protective effects of exercise against diabetic lung inflammation and fibrosis, the causal relationship cannot be definitively established. We did not employ macrophage-depletion models, and thus, whether macrophage polarization directly mediates the observed outcomes remains speculative. Additionally, the exercise groups exhibited significant reductions in adiposity. Considering that obesity itself is known to independently induce pulmonary inflammation and fibrosis [45,46], we cannot fully exclude the possibility that weight loss contributed to the observed pulmonary improvements. Future studies incorporating larger sample sizes and additional experimental groups (e.g., pair-fed or weight-matched controls) and employing genetic or pharmacological tools will help clarify the effects of different exercise modalities on diabetic lung disease and provide deeper mechanistic insights. Moreover, our study utilized only male mice, which limits the generalizability of the findings. Given that sex differences in both diabetes pathophysiology and exercise adaptation have been reported [85,86], future studies should include female subjects to determine whether the observed pulmonary benefits extend to both sexes. Finally, we did not measure blood glucose levels after exercise interventions. Fasting glucose was only used for initial group allocation. Previous studies have shown that HIIT can significantly lower fasting glucose and attenuate diabetic lung injury in rodents [25]. Therefore, future studies should include serial blood glucose assessments to determine whether improvements in pulmonary inflammation and fibrosis are mediated by enhanced glycemic control.

## 5. Conclusions

In this study, we found that T2DM induces oxidative stress in lung tissue, increases macrophage infiltration and promotes pro-inflammatory polarization, activates the TGF-β1/Smad signaling pathway, exacerbates EMT, and ultimately leads to pulmonary inflammation and fibrosis in mice. Swimming, resistance exercise, and HIIT can all alleviate these pathological changes by suppressing oxidative stress, reducing macrophage infiltration while promoting anti-inflammatory polarization, and inhibiting activation of the TGF-β1/Smad pathway and EMT progression, thereby improving T2DM-induced pulmonary inflammation and fibrosis. Among the three exercise modalities, swimming exhibited relatively stronger anti-inflammatory effects, whereas HIIT demonstrated a more pronounced inhibitory effect on the activation of fibrotic signaling pathways. These differences may, in part, be attributed to variations in exercise intensity. Therefore, for T2DM patients with the goal of improving pulmonary function, swimming or HIIT may be the most recommended exercise options. These findings further elucidate the beneficial effects and underlying mechanisms of exercise in alleviating diabetic lung complications.

## Figures and Tables

**Table 1:** List of primers used for RT-qPCR.

Gene	Forward Primer Sequences	Reverse Primer Sequences
*Nrf2*	5′-TCTTGGAGTAAGTCGAGAAGTGT-3′	5′-GTTGAAACTGAGCGAAAAAGGC-3′
*Hmox1*	5′-AAGCCGAGAATGCTGAGTTCA-3′	5′-GCCGTAGATATGGTACAAGGA-3′
*Keap1*	5′-TCGAAGGCATCCACCCTAAG-3′	5′-CTCGAACCACGCTGTCAATCT-3′
*Gpx3*	5′-CCTTTTAAGCAGTATGCAGGCA-3′	5′-CAAGCCAAATGGCCCAAGTT-3′
*Sod1*	5′-TATGGGGACAATACACAAGGCT-3′	5′-TATGGGGACAATACACAAGGCT-3′
*Nox2*	5′-TGATCCTGCTGCCAGTGTGTC-3′	5′-TGATCCTGCTGCCAGTGTGTC-3′
*TNFα*	5′-CCTGTAGCCCACGTCGTAG-3′	5′-GGGAGTAGACAAGGTACAACCC-3′
*IL6*	5′-TAGTCCTTCCTACCCCAATTTCC-3′	5′-TTGGTCCTTAGCCACTCCTTC-3′
*IL-1β*	5′-GAAATGCCACCTTTTGACAGTG-3′	5′-TGGATGCTCTCATCAGGACAG-3′
*IL-15*	5′-CATCCATCTCGTGCTACTTGTG-3′	5′-GCCTCTGTTTTAGGGAGACCT-3′
*NF-κB*	5′-GACACGACAGAATCCTCAGCATCC-3′	5′-GACACGACAGAATCCTCAGCATCC-3′
*CCL2*	5′-TAAAAACCTGGATCGGAACCAAA-3′	5′-GCATTAGCTTCAGATTTACGGGT-3′
*CCL5*	5′-GCTGCTTTGCCTACCTCTCC-3′	5′-TCGAGTGACAAACACGACTGC-3′
*IL-10*	5′-CTTACTGACTGGCATGAGGATCA-3′	5′-GCAGCTCTAGGAGCATGTGG-3′
*CD68*	5′-TGATCCTGCTGCCAGTGTGTC-3′	5′-TGATCCTGCTGCCAGTGTGTC-3′
*F4/80*	5-GCAAAAACTGGCAGTGGG-3′	5-GAATCTTGGCCAAGAAGAGAC-3′
*CD11c*	5′-GGTGCAAAGACCACCTTCAT-3′	5′-GACGTTTGAAGAAGCCAAGC-3′
*CD86*	5-GCAAAAACTGGCAGTGGG-3′	5-GCAAAAACTGGCAGTGGG-3′
*iNOS*	5′-GTTCTCAGCCCAACAATACAAGA-3′	5′-GTGGACGGGTCGATGTCAC-3′
*CD163*	5-GTTCCTGTCAAAGCTCGTGC-3′	5-GCAAAAACTGGCAGTGGG-3′
*CD206*	5-CTCTGTTCAGCTATTGGACGC-3	5-CGGAATTTCTGGGATTCAGCTTC-3′
*Arg1*	5′-CTCCAAGCCAAAGTCCTTAGAG-3′	5′-GGAGCTGTCATTAGGGACATCA-3′
*Col1a1*	5′-CTGGCGGTTCAGGTCCAAT-3′	5′-TTCCAGGCAATCCACGAGC-3′
*Col3a1*	5′-CTGTAACATGGAAACTGGGGAAA-3′	5′-CCATAGCTGAACTGAAAACCACC-3′
*MMP2*	5′-ACAAGTGGTCCGCGTAAAGT-3	5′-GTAAACAAGGCTTCATGGGGG-3′
*MMP9*	5′-GGACCCGAAGCGGACATTG-3′	5′-CGTCGTCGAAATGGGCATCT-3′
*Fibronectin*	5′-ATGTGGACCCCTCCTGATAGT-3′	5′-GCCCAGTGATTTCAGCAAAGG-3′
*TGF-β*	5′-CTTCAATACGTCAGACATTCGGG-3′	5′-GTAACGCCAGGAATTGTTGCTA-3′
*α-SMA*	5′-GTCCCAGACATCAGGGAGTAA-3′	5′-TCGGATACTTCAGCGTCAGGA-3′
*E-cadherin*	5′-CAGTTCCGAGGTCTACACCTT-3′	5′-TGAATCGGGAGTCTTCCGAAAA-3′
*N-cadherin*	5′-AGGCTTCTGGTGAAATTGCAT-3′	5′-GTCCACCTTGAAATCTGCTGG-3′
*Vimentin*	5′-TCCACACGCACCTACAGTCT-3′	5′-CCGAGGACCGGGTCACATA-3′
*β-actin*	5′-CGTGCGTGACATCAAAGAGAA-3′	5′-GCTCGTTGCCAATAGTGATGA-3′

## Data Availability

The original contributions presented in the study are included in the article; further inquiries can be directed to the corresponding authors.
